# Crystal structure of hexa­kis­(urea-κ*O*)chromium(III) dichromate bromide monohydrate from synchrotron X-ray data

**DOI:** 10.1107/S2056989015019258

**Published:** 2015-10-17

**Authors:** Dohyun Moon, Shinnosuke Tanaka, Takashiro Akitsu, Jong-Ha Choi

**Affiliations:** a Pohang Accelerator Laboratory, POSTECH, Pohang 37673, Republic of Korea; b Department of Chemistry, Tokyo University of Science, 1-3 Kagurazaka, Shinjuku-ku, Tokyo 162-8601, Japan; c Department of Chemistry, Andong National University, Andong 36729, Republic of Korea

**Keywords:** crystal structure, chromium(III) complex, urea ligand, bromide salt, hydrogen bonding, synchrotron data

## Abstract

The title bromide salt, [Cr{CO(NH_2_)_2_}_6_](Cr_2_O_7_)Br·H_2_O, is isotypic to the corresponding chloride salt. Within the complex cation, the Cr^III^ atom is coordinated by six O atoms of six urea ligands, displaying a slightly distorted octa­hedral coordination environment. The Cr—O bond lengths involving the urea ligands are in the range 1.9534 (13)–1.9776 (12) Å. The Cr_2_O_7_^2−^ anion has a nearly staggered conformation, with a bridging angle of 130.26 (10)°. The individual components are arranged in rows extending parallel to [100]. The Br^−^ anion links the complex cation, as well as the solvent water mol­ecule, through N—H⋯Br and O—H⋯Br hydrogen-bonding inter­actions. The supra­molecular architecture also includes N—H⋯O and O—H⋯O hydrogen bonding between urea N—H and water O—H donor groups and the O atoms of the Cr_2_O_7_^2−^ anion as acceptor atoms, leading to a three-dimensional network structure.

## Abstract 2

The title compound, [Cr(urea)_6_](Cr_2_O_7_)Br·H_2_O, is isotypic with the corresponding chloride salt and consists of discrete [Cr(urea)_6_]^3+^ cations, staggered Cr_2_O_7_^4−^ and Br^−^ anions and water solvent mol­ecules that are held together by an intricate three-dimensional network of hydrogen-bonding inter­actions.

## Chemical context   

Counter-ionic species in coordination compounds play important roles in chemistry, pharmacy, mol­ecular assembly, biology and catalysis, as well as contributing significantly to environmental pollution; however, their binding characteristics have not received much recognition (Martínez-Máñez & Sancenón, 2003 ▸; Fabbrizzi & Poggi, 2013 ▸). The study of the anion or cation effect in octa­hedral metal complexes may be expected to yield a great variety of new structures and properties of both chemical and biological significance. Chromium is usually found in trivalent and hexa­valent oxidation states in soil, ground water and seawater (Cespon-Romero *et al.*, 1996 ▸). Cr^III^ is an essential element in mammals for maintaining efficient glucose, lipid and protein metabolism. On the other hand, Cr^VI^ is toxic and recognized as a carcinogen to humans and wildlife. The dichromate ion is environmentally important due to its high toxicity (Yusof & Malek, 2009 ▸) and its use in many industrial processes (Goyal *et al.*, 2003 ▸). Recently, the ionic reactions between hexa­ureachromium(III) and inorganic oxoanions (such as Cr_2_O_7_^2−^ or CrO_4_^2−^) in aqueous solution have been investigated. It was found that [Cr(urea)_6_]^3+^ is suitable to target these oxoanions (Bala *et al.*, 2013 ▸). Previously, the crystal structure of [Cr(urea)_6_](Cr_2_O_7_)Cl·H_2_O has been reported (Bondar *et al.*, 1984 ▸). This complex crystallizes in the monoclinic space group *P*2/*n* with four formula units in a cell of dimensions *a* = 13.782 (2), *b* = 10.393 (1), *c* = 17.794 (3) Å and β = 94. 86 (2)°. Within our broader study of Cr^III^ complexes as industrial materials (Choi & Lee, 2009 ▸; Choi & Moon, 2014 ▸; Moon & Choi, 2015 ▸), we report herein the preparation and crystal structure of [Cr(urea)_6_](Cr_2_O_7_)Br·H_2_O, (I).
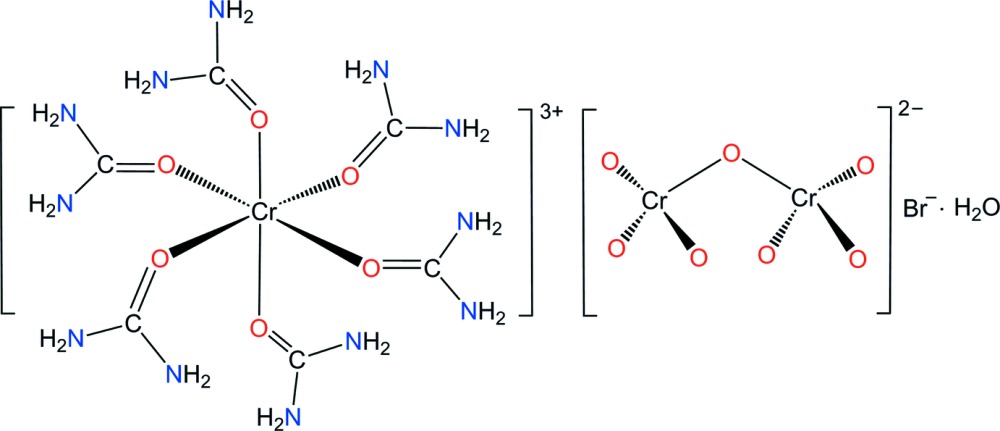


## Structural commentary   

**Figure 1:**
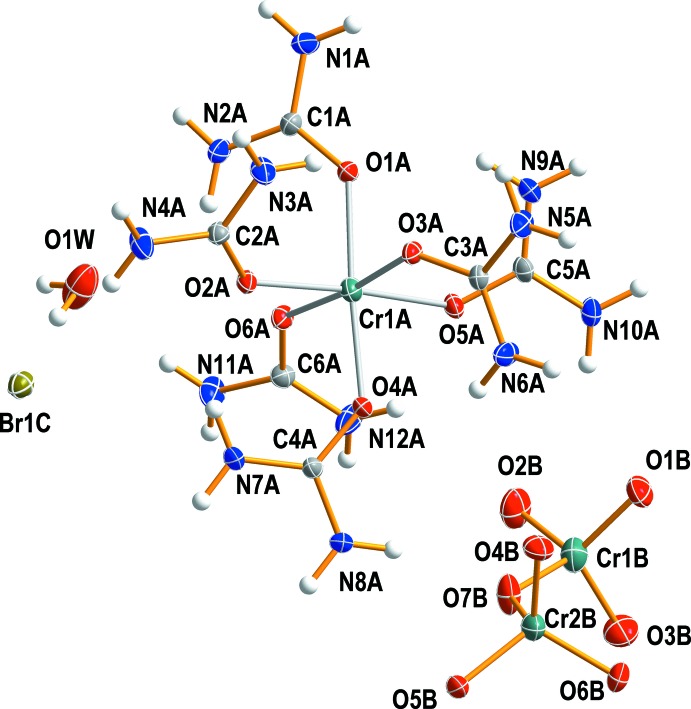
The mol­ecular structures of the components in compound (I), showing the atom-numbering scheme. Displacement ellipsoids are drawn at the 50% probability level.

In order to check if compound (I) is isotypic to [Cr(urea)_6_](Cr_2_O_7_)Cl·H_2_O investigated previously (Bondar *et al.*, 1984 ▸), a single-crystal X-ray structure determination was performed on the basis of synchrotron data. Compound (I) consists of the isolated complex cation [Cr(urea)_6_]^3+^, together with Cr_2_O_7_^2−^ and Br^−^ counter-ions and a solvent water mol­ecule. Comparison of the space-group type, metrics and the arrangement of the mol­ecular components reveal (I) to be isotypic to the corresponding chloride salt. An ellipsoid plot of the mol­ecular components of compound (I) is depicted in Fig. 1 ▸.

The Cr^III^ ion is coordinated by six urea ligands through oxygen atoms with Cr*A*—O*A* bond lengths ranging from 1.9534 (13) to 1.9776 (12) Å, and with O*A*—Cr*A*—O*A* bond angles in the range 85.10 (5)–92.95 (5)°. The Cr*A*—O*A* bond lengths involving the urea ligand are in good agreement with the value of 1.9630 (17) Å for [Cr(urea)_6_](BF_4_)_3_ (Górska *et al.*, 2014 ▸). They are also comparable with the corresponding lengths determined for *trans*-[Cr(nic-*O*)_2_(cyclam)]ClO_4_ (cyclam = 1,4,8,11-tetra­aza­cyclo­tetra­decane; nic-*O* = *O*-coordinating nicotinate; Choi, 2009 ▸), *cis*-[Cr(ox)(cyclam)]ClO_4_ (ox = oxalate; Choi *et al.*, 2004*a* ▸), *cis*-[Cr(acac)(cyclam)](ClO_4_)_2_·0.5H_2_O (acac = acetyl­acetonate; Subhan *et al.*, 2011 ▸), *cis*-[Cr(ONO)_2_(cyclam)]NO_2_ (Choi *et al.*, 2004*b* ▸) or *cis*-[Cr(edda)(acac)] (edda = ethyl­enedi­amine-*N,N*’-di­acetate; Choi *et al.*, 2012 ▸). The *trans* O1*A*—Cr1*A*—O4*A*, O3*A*—Cr1*A*—O6*A* and O2*A*—Cr1*A*—O5*A* bond angles are 176.27 (5)°, 173.94 (5)°, and 175.89 (5)°, respectively. The bond lengths within the urea ligand are in the ranges of 1.263 (2)–1.276 (2) and 1.316 (2)–1.328 (2) Å for C=O and C—N bonds, respectively. The C=O bonds are slightly longer than that in free non-coordinating urea (Guth *et al.*, 1980 ▸). The isolated Cr_2_O_7_^2−^ and Br^−^ anions remain outside the coordination sphere of the cation.

It is of inter­est to compare the conformation of Cr_2_O_7_^2−^ with that found in other ionic crystals. In the structure of compound (I) it is in a nearly staggered conformation, whereas in K_2_Cr_2_O_7_, the tetra­hedral CrO_4_ groups are in an almost eclipsed conformation (Brandon & Brown, 1968 ▸). As expected, the two bridging Cr*B*—O*B* bonds of 1.7643 (18) and 1.8011 (17) Å are longer than the terminal Cr*B*—O*B* bonds that are in the range of 1.6014 (16)–1.6299 (14) Å. The Cr1*B*—O7*B*—Cr2*B* bridging angle in the complex anion is 130.26 (10)°. The O*B*—Cr*B*—O*B* bond angles in the two tetra­hedral CrO_4_ groups are between 105.21 (8) and 110.98 (10)°, indicating slight angular distortions.

It is confirmed that the [Cr(urea)_6_]^3+^ moiety in compound (I) may be used as a potential receptor for Cr_2_O_7_^2−^ anions due to its high positive charge and the large number of hydrogen-bond donor groups of its six urea ligands.

## Supra­molecular features   

**Figure 2:**
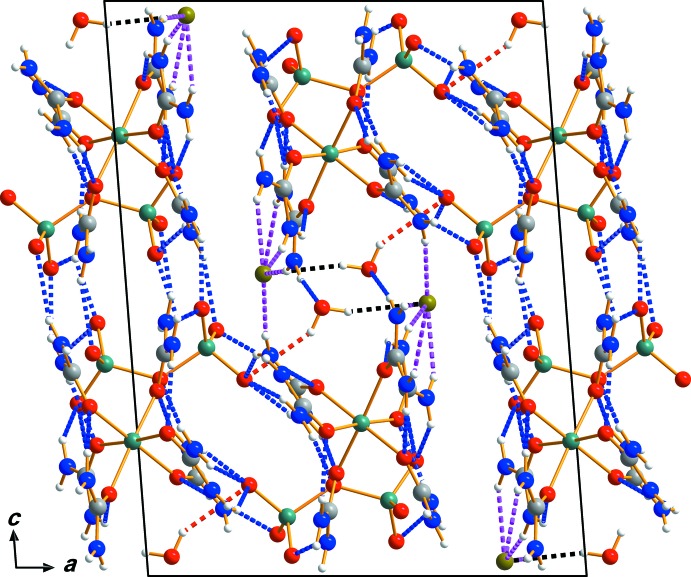
The crystal packing of compound (I), viewed perpendicular to the *ac* plane. Dashed lines represent hydrogen-bonding inter­actions of the types N—H⋯O (blue), N—H⋯Br (pink), O—H⋯O (red) and O—H⋯Br (black).

The individual mol­ecular or ionic components of (I) are arranged in rows extending parallel to [100]. The packing in the crystal structure of (I) involves not only hydrogen bonds of the type N—H⋯O between urea amino donor groups and the O acceptor atoms of carbonyl groups, the water mol­ecule, or the Cr_2_O_7_^2−^ anion, but also N—H⋯Br hydrogen bonding between the urea amino groups and the Br^−^ anion (Table 1 ▸). O—H⋯Br inter­actions involving the water mol­ecule are also observed. All these inter­actions are responsible for the formation of an intricate three-dimensional hydrogen-bonded network in (I) (Fig. 2 ▸).

## Synthesis and crystallization   

All chemicals were reagent-grade materials and used without further purification. Chromium(III) tribromide hexa­hydrate was obtained from Aldrich Chemical Co. and used as supplied. [Cr(urea)_6_]Br_3_·3H_2_O was used as the starting material and was prepared according to literature procedures (Brauer, 1965 ▸), except that chromium(III) tribromide hexa­hydrate was used in place of chromium(III) trichloride hexa­hydrate (Flint & Palacio, 1979 ▸). A 0.5 g sample of [Cr(urea)_6_]Br_3_·3H_2_O was dissolved in 20 mL of water. Potassium dichromate (0.22 g), dissolved in 10 mL of water, was added to this solution. The mixture was refluxed at 353 K for 10 min and then cooled to room temperature. Green crystals of (I) suitable for X-ray structure analysis formed overnight. These were collected by filtration, washed with 2-propanol, and air dried. Yield: 65%. Elemental analysis calculated for [Cr{CO(NH_2_)_2_}_6_](Cr_2_O_7_)Br·H_2_O: C, 9.92; H, 3.61; N, 23.14%; found: C, 10.32; H, 3.08; N, 23.38%.

## Refinement   

Crystal data, data collection and structure refinement details are summarized in Table 2 ▸. H atoms bound to nitro­gen were placed at calculated positions and treated as riding on their parent atoms, with N—H = 0.87 Å and *U*_iso_(H) = 1.2*U*_eq_(N). H atoms of the solvent water mol­ecule were found from difference maps and refined with *U*_iso_(H) = 1.2*U*_eq_(O) and restrained to O—H = 0.84 (1) and H⋯H = 1.36 (2) Å.

## Supplementary Material

Crystal structure: contains datablock(s) I. DOI: 10.1107/S2056989015019258/wm5225sup1.cif

Structure factors: contains datablock(s) I. DOI: 10.1107/S2056989015019258/wm5225Isup2.hkl

CCDC reference: 1430688

Additional supporting information:  crystallographic information; 3D view; checkCIF report

## Figures and Tables

**Table 1:** Hydrogen-bond geometry (, )

*D*H*A*	*D*H	H*A*	*D* *A*	*D*H*A*
N1*A*H1*A*1O2*B* ^i^	0.87	2.42	3.051(3)	129
N1*A*H1*A*2Br1*C* ^ii^	0.87	2.60	3.4031(18)	155
N2*A*H2*A*1O6*A*	0.87	2.20	2.890(2)	136
N2*A*H2*A*1O1*W*	0.87	2.58	3.230(3)	132
N2*A*H2*A*2Br1*C* ^ii^	0.87	2.73	3.5121(17)	150
N3*A*H3*A*1O3*A*	0.87	2.23	2.866(2)	130
N3*A*H3*A*2O5*B* ^iii^	0.87	2.21	2.972(2)	147
N4*A*H4*A*1Br1*C*	0.87	2.55	3.4070(18)	171
N4*A*H4*A*2O5*B* ^iii^	0.87	2.11	2.900(2)	151
N5*A*H5*A*1O6*B* ^i^	0.87	2.52	3.174(2)	133
N5*A*H5*A*2O3*B* ^iv^	0.87	2.33	3.108(2)	150
N6*A*H6*A*1O4*A*	0.87	2.17	2.914(2)	143
N6*A*H6*A*2O1*B* ^iv^	0.87	2.15	2.920(2)	147
N7*A*H7*A*1O2*A*	0.87	2.12	2.865(2)	143
N7*A*H7*A*2O4*B* ^v^	0.87	2.24	3.092(2)	168
N8*A*H8*A*1O7*B*	0.87	2.03	2.883(2)	166
N8*A*H8*A*2O5*B* ^v^	0.87	2.04	2.875(2)	162
N9*A*H9*A*1O1*A*	0.87	2.22	2.954(2)	141
N9*A*H9*A*2O6*B* ^iv^	0.87	2.21	2.997(2)	150
N10*A*H10*A*O1*B*	0.87	2.29	3.028(2)	142
N10*A*H10*B*O4*B* ^iv^	0.87	2.39	3.184(2)	151
N11*A*H11*A*O1*W*	0.87	2.04	2.892(3)	167
N11*A*H11*B*Br1*C* ^vi^	0.87	2.57	3.4031(18)	162
N12*A*H12*A*O5*A*	0.87	2.27	2.982(2)	140
N12*A*H12*A*O2*B*	0.87	2.54	3.051(2)	118
N12*A*H12*B*Br1*C* ^vi^	0.87	2.85	3.6242(18)	149
O1*W*H1*O*1Br1*C*	0.85(1)	2.51(1)	3.332(2)	163(3)
O1*W*H2*O*1O5*B* ^vii^	0.84(1)	2.57(2)	3.315(3)	149(3)

Symmetry codes: (i) 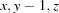; (ii) 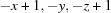; (iii) 
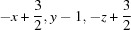
; (iv) 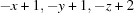; (v) 
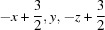
; (vi) 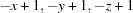; (vii) 
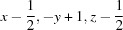
.

**Table 2:** Experimental details

Crystal data	
Chemical formula	[Cr(CH_4_N_2_O)_6_](Cr_2_O_7_)BrH_2_O
*M*_r_	726.30
Crystal system, space group	Monoclinic, *P*2/*n*
Temperature (K)	243
*a*, *b*, *c* ()	13.774(3), 10.474(2), 18.123(4)
()	94.37(3)
*V* (^3^)	2607.0(9)
*Z*	4
Radiation type	Synchrotron, = 0.620
(mm^1^)	1.97
Crystal size (mm)	0.17 0.08 0.04

Data collection	
Diffractometer	ADSC Q210 CCD area detector
Absorption correction	Empirical (using intensity measurements) (*HKL3000sm *SCALEPACK**; Otwinowski Minor, 1997 ▸)
*T*_min_, *T*_max_	0.727, 0.920
No. of measured, independent and observed [*I* > 2(*I*)] reflections	27665, 7308, 5831
*R*_int_	0.036
(sin /)_max_ (^1^)	0.694

Refinement	
*R*[*F* ^2^ > 2(*F* ^2^)], *wR*(*F* ^2^), *S*	0.031, 0.091, 1.04
No. of reflections	7308
No. of parameters	331
No. of restraints	3
H-atom treatment	H atoms treated by a mixture of independent and constrained refinement
_max_, _min_ (e ^3^)	1.02, 1.25

Computer programs: *PAL ADSC Quantum-210 ADX* (Arvai Nielsen, 1983 ▸), *HKL3000sm* (Otwinowski Minor, 1997 ▸), *SHELXT* (Sheldrick, 2015*a* ▸), *SHELXL2014* (Sheldrick, 2015*b* ▸), *DIAMOND* (Putz Brandenburg, 2007 ▸) and *publCIF* (Westrip, 2010 ▸).

## supplementary crystallographic information

### Crystal data

**Table:**

[Cr(CH_4_N_2_O)_6_](Cr_2_O_7_)Br·H_2_O	*F*(000) = 1460
*M**_r_* = 726.30	*D*_x_ = 1.850 Mg m^−^^3^
Monoclinic, *P*2/*n*	Synchrotron radiation, λ = 0.620 Å
*a* = 13.774 (3) Å	Cell parameters from 102484 reflections
*b* = 10.474 (2) Å	θ = 0.4–33.6°
*c* = 18.123 (4) Å	µ = 1.97 mm^−^^1^
β = 94.37 (3)°	*T* = 243 K
*V* = 2607.0 (9) Å^3^	Block, green
*Z* = 4	0.17 × 0.08 × 0.04 mm

### Data collection

**Table:**

ADSC Q210 CCD area-detector diffractometer	5831 reflections with *I* > 2σ(*I*)
Radiation source: PLSII 2D bending magnet	*R*_int_ = 0.036
ω scans	θ_max_ = 25.5°, θ_min_ = 1.6°
Absorption correction: empirical (using intensity measurements) (*HKL3000sm *SCALEPACK**; Otwinowski & Minor, 1997)	*h* = −19→19
*T*_min_ = 0.727, *T*_max_ = 0.920	*k* = −14→14
27665 measured reflections	*l* = −25→25
7308 independent reflections	

### Refinement

**Table:**

Refinement on *F*^2^	3 restraints
Least-squares matrix: full	Hydrogen site location: mixed
*R*[*F*^2^ > 2σ(*F*^2^)] = 0.031	H atoms treated by a mixture of independent and constrained refinement
*wR*(*F*^2^) = 0.091	*w* = 1/[σ^2^(*F*_o_^2^) + (0.0607*P*)^2^] where *P* = (*F*_o_^2^ + 2*F*_c_^2^)/3
*S* = 1.03	(Δ/σ)_max_ = 0.001
7308 reflections	Δρ_max_ = 1.01 e Å^−^^3^
331 parameters	Δρ_min_ = −1.25 e Å^−^^3^

### Special details

**Table:**

Geometry. All e.s.d.'s (except the e.s.d. in the dihedral angle between two l.s. planes) are estimated using the full covariance matrix. The cell e.s.d.'s are taken into account individually in the estimation of e.s.d.'s in distances, angles and torsion angles; correlations between e.s.d.'s in cell parameters are only used when they are defined by crystal symmetry. An approximate (isotropic) treatment of cell e.s.d.'s is used for estimating e.s.d.'s involving l.s. planes.

### Fractional atomic coordinates and isotropic or equivalent isotropic displacement parameters (Å^2^)

**Table:**

	*x*	*y*	*z*	*U*_iso_*/*U*_eq_	
Cr1A	0.48784 (2)	0.24358 (2)	0.73428 (2)	0.01814 (7)	
O1A	0.40481 (9)	0.09045 (11)	0.72718 (6)	0.0269 (3)	
N1A	0.33471 (14)	−0.09246 (16)	0.69010 (10)	0.0418 (4)	
H1A1	0.3286	−0.1131	0.7360	0.050*	
H1A2	0.3140	−0.1441	0.6547	0.050*	
N2A	0.38377 (13)	0.04656 (16)	0.60403 (9)	0.0365 (4)	
H2A1	0.4103	0.1185	0.5925	0.044*	
H2A2	0.3627	−0.0062	0.5694	0.044*	
C1A	0.37546 (13)	0.01753 (16)	0.67403 (9)	0.0251 (3)	
O2A	0.58364 (9)	0.16617 (11)	0.67175 (6)	0.0247 (2)	
N3A	0.61554 (13)	−0.03008 (15)	0.72164 (9)	0.0363 (4)	
H3A1	0.5774	−0.0167	0.7569	0.044*	
H3A2	0.6464	−0.1023	0.7194	0.044*	
N4A	0.68354 (14)	0.03653 (17)	0.61675 (10)	0.0432 (4)	
H4A1	0.6904	0.0939	0.5828	0.052*	
H4A2	0.7140	−0.0360	0.6151	0.052*	
C2A	0.62668 (13)	0.05977 (16)	0.67111 (9)	0.0247 (3)	
O3A	0.55288 (9)	0.15949 (11)	0.82167 (6)	0.0242 (2)	
N5A	0.58535 (13)	0.11285 (15)	0.94126 (8)	0.0351 (4)	
H5A1	0.5743	0.0326	0.9316	0.042*	
H5A2	0.6018	0.1370	0.9864	0.042*	
N6A	0.59470 (13)	0.31974 (15)	0.90232 (9)	0.0352 (4)	
H6A1	0.5899	0.3762	0.8670	0.042*	
H6A2	0.6111	0.3434	0.9476	0.042*	
C3A	0.57714 (12)	0.19830 (17)	0.88725 (9)	0.0228 (3)	
O4A	0.56802 (9)	0.39845 (11)	0.74788 (6)	0.0244 (2)	
N7A	0.66023 (14)	0.41622 (16)	0.64918 (10)	0.0432 (5)	
H7A1	0.6546	0.3351	0.6395	0.052*	
H7A2	0.6937	0.4649	0.6217	0.052*	
N8A	0.62761 (13)	0.58826 (15)	0.72028 (9)	0.0366 (4)	
H8A1	0.6004	0.6213	0.7576	0.044*	
H8A2	0.6613	0.6363	0.6925	0.044*	
C4A	0.61789 (12)	0.46555 (16)	0.70582 (9)	0.0228 (3)	
O5A	0.39560 (9)	0.33272 (11)	0.79418 (6)	0.0268 (3)	
N9A	0.34380 (13)	0.17981 (16)	0.87118 (9)	0.0365 (4)	
H9A1	0.3558	0.1195	0.8401	0.044*	
H9A2	0.3203	0.1609	0.9130	0.044*	
N10A	0.34220 (13)	0.39063 (16)	0.90220 (9)	0.0394 (4)	
H10A	0.3531	0.4702	0.8919	0.047*	
H10B	0.3187	0.3706	0.9439	0.047*	
C5A	0.36129 (12)	0.30050 (18)	0.85473 (9)	0.0249 (3)	
O6A	0.42481 (9)	0.31007 (11)	0.64197 (6)	0.0267 (3)	
N11A	0.38747 (13)	0.44107 (17)	0.54758 (9)	0.0410 (4)	
H11A	0.4000	0.3802	0.5171	0.049*	
H11B	0.3686	0.5155	0.5306	0.049*	
N12A	0.37717 (14)	0.51221 (16)	0.66616 (9)	0.0407 (4)	
H12A	0.3828	0.4985	0.7136	0.049*	
H12B	0.3584	0.5866	0.6491	0.049*	
C6A	0.39707 (13)	0.42046 (16)	0.62008 (9)	0.0263 (3)	
Cr1B	0.43809 (2)	0.74013 (3)	0.86870 (2)	0.02751 (8)	
Cr2B	0.67274 (2)	0.73371 (2)	0.89720 (2)	0.02405 (7)	
O1B	0.42792 (12)	0.65095 (15)	0.93969 (8)	0.0452 (4)	
O2B	0.36945 (13)	0.6882 (2)	0.79973 (9)	0.0586 (5)	
O3B	0.41248 (13)	0.88526 (15)	0.88712 (10)	0.0553 (4)	
O4B	0.69792 (12)	0.59234 (12)	0.92738 (9)	0.0425 (3)	
O5B	0.75382 (11)	0.78025 (13)	0.84199 (8)	0.0350 (3)	
O6B	0.66869 (12)	0.83207 (14)	0.96458 (8)	0.0446 (4)	
O7B	0.56137 (12)	0.73256 (16)	0.84224 (9)	0.0475 (4)	
Br1C	0.68438 (2)	0.24957 (2)	0.47474 (2)	0.03324 (7)	
O1W	0.44307 (16)	0.2183 (3)	0.46645 (14)	0.0752 (7)	
H1O1	0.5018 (9)	0.236 (3)	0.460 (2)	0.090*	
H2O1	0.414 (2)	0.224 (3)	0.4242 (10)	0.090*	

### Atomic displacement parameters (Å^2^)

**Table:**

	*U*^11^	*U*^22^	*U*^33^	*U*^12^	*U*^13^	*U*^23^
Cr1A	0.02397 (14)	0.01721 (12)	0.01352 (12)	−0.00045 (9)	0.00324 (9)	0.00064 (8)
O1A	0.0347 (7)	0.0256 (6)	0.0205 (6)	−0.0087 (5)	0.0029 (5)	−0.0020 (4)
N1A	0.0600 (12)	0.0324 (9)	0.0328 (9)	−0.0201 (8)	0.0028 (8)	−0.0024 (7)
N2A	0.0584 (11)	0.0293 (8)	0.0217 (7)	−0.0092 (7)	0.0016 (7)	−0.0050 (6)
C1A	0.0267 (8)	0.0252 (8)	0.0233 (8)	−0.0013 (6)	0.0011 (6)	−0.0021 (6)
O2A	0.0324 (6)	0.0207 (5)	0.0219 (5)	0.0047 (5)	0.0082 (5)	0.0012 (4)
N3A	0.0517 (10)	0.0254 (7)	0.0334 (8)	0.0121 (7)	0.0141 (7)	0.0084 (6)
N4A	0.0590 (11)	0.0328 (8)	0.0412 (10)	0.0212 (8)	0.0266 (8)	0.0096 (7)
C2A	0.0294 (9)	0.0221 (7)	0.0225 (8)	0.0029 (6)	0.0015 (6)	−0.0001 (6)
O3A	0.0340 (7)	0.0222 (5)	0.0160 (5)	0.0008 (5)	−0.0004 (4)	0.0003 (4)
N5A	0.0553 (11)	0.0303 (8)	0.0189 (7)	0.0038 (7)	−0.0019 (7)	0.0039 (6)
N6A	0.0535 (10)	0.0297 (8)	0.0211 (7)	−0.0090 (7)	−0.0058 (7)	−0.0008 (6)
C3A	0.0228 (8)	0.0271 (8)	0.0185 (7)	0.0025 (6)	0.0020 (6)	0.0002 (6)
O4A	0.0318 (6)	0.0227 (6)	0.0195 (5)	−0.0069 (5)	0.0071 (5)	−0.0003 (4)
N7A	0.0638 (12)	0.0300 (8)	0.0403 (10)	−0.0118 (8)	0.0336 (9)	−0.0061 (7)
N8A	0.0521 (10)	0.0250 (7)	0.0352 (8)	−0.0146 (7)	0.0194 (8)	−0.0063 (6)
C4A	0.0260 (8)	0.0240 (7)	0.0186 (7)	−0.0051 (6)	0.0024 (6)	0.0000 (6)
O5A	0.0320 (7)	0.0264 (6)	0.0234 (6)	0.0031 (5)	0.0108 (5)	0.0025 (5)
N9A	0.0468 (10)	0.0337 (8)	0.0312 (8)	−0.0092 (7)	0.0179 (7)	0.0007 (7)
N10A	0.0522 (11)	0.0358 (9)	0.0330 (9)	0.0025 (8)	0.0224 (8)	−0.0046 (7)
C5A	0.0200 (8)	0.0314 (9)	0.0239 (8)	−0.0003 (6)	0.0060 (6)	−0.0002 (6)
O6A	0.0372 (7)	0.0231 (6)	0.0192 (5)	0.0041 (5)	−0.0018 (5)	0.0018 (4)
N11A	0.0637 (12)	0.0345 (9)	0.0242 (8)	0.0140 (8)	0.0005 (8)	0.0082 (7)
N12A	0.0614 (12)	0.0286 (8)	0.0307 (9)	0.0152 (8)	−0.0061 (8)	−0.0029 (6)
C6A	0.0281 (9)	0.0253 (8)	0.0248 (8)	0.0009 (7)	−0.0026 (7)	0.0024 (6)
Cr1B	0.03041 (16)	0.03174 (16)	0.02060 (14)	0.00408 (11)	0.00341 (11)	−0.00030 (10)
Cr2B	0.03046 (15)	0.02096 (13)	0.02202 (14)	0.00009 (10)	0.01038 (11)	−0.00170 (10)
O1B	0.0603 (10)	0.0450 (9)	0.0315 (7)	0.0022 (7)	0.0112 (7)	0.0076 (6)
O2B	0.0538 (11)	0.0844 (13)	0.0358 (9)	−0.0078 (10)	−0.0086 (7)	−0.0112 (9)
O3B	0.0664 (12)	0.0354 (8)	0.0655 (11)	0.0140 (8)	0.0136 (9)	0.0039 (8)
O4B	0.0544 (9)	0.0253 (7)	0.0493 (9)	0.0019 (6)	0.0139 (7)	0.0074 (6)
O5B	0.0439 (8)	0.0273 (6)	0.0364 (7)	−0.0072 (6)	0.0200 (6)	−0.0046 (5)
O6B	0.0658 (10)	0.0378 (8)	0.0321 (7)	−0.0006 (7)	0.0166 (7)	−0.0119 (6)
O7B	0.0365 (9)	0.0763 (12)	0.0306 (8)	0.0079 (7)	0.0076 (6)	−0.0056 (7)
Br1C	0.04246 (12)	0.02884 (10)	0.02779 (11)	0.00280 (7)	−0.00149 (8)	−0.00060 (6)
O1W	0.0493 (12)	0.0971 (16)	0.0789 (16)	−0.0032 (12)	0.0040 (11)	−0.0419 (14)

### Geometric parameters (Å, º)

**Table:**

Cr1A—O6A	1.9534 (13)	N7A—H7A1	0.8700
Cr1A—O4A	1.9672 (12)	N7A—H7A2	0.8700
Cr1A—O3A	1.9674 (12)	N8A—C4A	1.316 (2)
Cr1A—O5A	1.9683 (12)	N8A—H8A1	0.8700
Cr1A—O1A	1.9685 (12)	N8A—H8A2	0.8700
Cr1A—O2A	1.9776 (12)	O5A—C5A	1.273 (2)
O1A—C1A	1.271 (2)	N9A—C5A	1.325 (3)
N1A—C1A	1.324 (2)	N9A—H9A1	0.8700
N1A—H1A1	0.8700	N9A—H9A2	0.8700
N1A—H1A2	0.8700	N10A—C5A	1.317 (2)
N2A—C1A	1.318 (2)	N10A—H10A	0.8700
N2A—H2A1	0.8700	N10A—H10B	0.8700
N2A—H2A2	0.8700	O6A—C6A	1.272 (2)
O2A—C2A	1.263 (2)	N11A—C6A	1.328 (2)
N3A—C2A	1.330 (2)	N11A—H11A	0.8700
N3A—H3A1	0.8700	N11A—H11B	0.8700
N3A—H3A2	0.8700	N12A—C6A	1.316 (2)
N4A—C2A	1.327 (2)	N12A—H12A	0.8700
N4A—H4A1	0.8700	N12A—H12B	0.8700
N4A—H4A2	0.8700	Cr1B—O3B	1.6014 (16)
O3A—C3A	1.2763 (19)	Cr1B—O2B	1.6036 (17)
N5A—C3A	1.325 (2)	Cr1B—O1B	1.6045 (15)
N5A—H5A1	0.8700	Cr1B—O7B	1.8011 (17)
N5A—H5A2	0.8700	Cr2B—O6B	1.6018 (14)
N6A—C3A	1.319 (2)	Cr2B—O4B	1.6071 (14)
N6A—H6A1	0.8700	Cr2B—O5B	1.6299 (14)
N6A—H6A2	0.8700	Cr2B—O7B	1.7643 (18)
O4A—C4A	1.2753 (19)	O1W—H1O1	0.847 (10)
N7A—C4A	1.324 (2)	O1W—H2O1	0.840 (10)
			
O6A—Cr1A—O4A	91.26 (5)	C4A—O4A—Cr1A	134.62 (11)
O6A—Cr1A—O3A	173.94 (5)	C4A—N7A—H7A1	120.0
O4A—Cr1A—O3A	92.95 (5)	C4A—N7A—H7A2	120.0
O6A—Cr1A—O5A	92.28 (5)	H7A1—N7A—H7A2	120.0
O4A—Cr1A—O5A	85.39 (5)	C4A—N8A—H8A1	120.0
O3A—Cr1A—O5A	92.40 (5)	C4A—N8A—H8A2	120.0
O6A—Cr1A—O1A	90.94 (5)	H8A1—N8A—H8A2	120.0
O4A—Cr1A—O1A	176.27 (5)	O4A—C4A—N8A	118.05 (15)
O3A—Cr1A—O1A	85.10 (5)	O4A—C4A—N7A	122.52 (15)
O5A—Cr1A—O1A	91.51 (5)	N8A—C4A—N7A	119.42 (16)
O6A—Cr1A—O2A	85.86 (5)	C5A—O5A—Cr1A	130.34 (11)
O4A—Cr1A—O2A	90.98 (5)	C5A—N9A—H9A1	120.0
O3A—Cr1A—O2A	89.71 (5)	C5A—N9A—H9A2	120.0
O5A—Cr1A—O2A	175.89 (5)	H9A1—N9A—H9A2	120.0
O1A—Cr1A—O2A	92.18 (5)	C5A—N10A—H10A	120.0
C1A—O1A—Cr1A	133.54 (11)	C5A—N10A—H10B	120.0
C1A—N1A—H1A1	120.0	H10A—N10A—H10B	120.0
C1A—N1A—H1A2	120.0	O5A—C5A—N10A	118.60 (17)
H1A1—N1A—H1A2	120.0	O5A—C5A—N9A	122.19 (16)
C1A—N2A—H2A1	120.0	N10A—C5A—N9A	119.21 (16)
C1A—N2A—H2A2	120.0	C6A—O6A—Cr1A	134.00 (11)
H2A1—N2A—H2A2	120.0	C6A—N11A—H11A	120.0
O1A—C1A—N2A	123.02 (16)	C6A—N11A—H11B	120.0
O1A—C1A—N1A	118.16 (16)	H11A—N11A—H11B	120.0
N2A—C1A—N1A	118.82 (16)	C6A—N12A—H12A	120.0
C2A—O2A—Cr1A	134.53 (11)	C6A—N12A—H12B	120.0
C2A—N3A—H3A1	120.0	H12A—N12A—H12B	120.0
C2A—N3A—H3A2	120.0	O6A—C6A—N12A	122.57 (16)
H3A1—N3A—H3A2	120.0	O6A—C6A—N11A	117.47 (16)
C2A—N4A—H4A1	120.0	N12A—C6A—N11A	119.95 (17)
C2A—N4A—H4A2	120.0	O3B—Cr1B—O2B	110.98 (10)
H4A1—N4A—H4A2	120.0	O3B—Cr1B—O1B	110.57 (9)
O2A—C2A—N4A	118.21 (16)	O2B—Cr1B—O1B	110.20 (10)
O2A—C2A—N3A	122.64 (16)	O3B—Cr1B—O7B	108.93 (8)
N4A—C2A—N3A	119.13 (16)	O2B—Cr1B—O7B	106.82 (9)
C3A—O3A—Cr1A	132.68 (11)	O1B—Cr1B—O7B	109.24 (9)
C3A—N5A—H5A1	120.0	O6B—Cr2B—O4B	110.63 (8)
C3A—N5A—H5A2	120.0	O6B—Cr2B—O5B	109.93 (8)
H5A1—N5A—H5A2	120.0	O4B—Cr2B—O5B	110.14 (8)
C3A—N6A—H6A1	120.0	O6B—Cr2B—O7B	110.82 (8)
C3A—N6A—H6A2	120.0	O4B—Cr2B—O7B	109.98 (8)
H6A1—N6A—H6A2	120.0	O5B—Cr2B—O7B	105.21 (8)
O3A—C3A—N6A	122.05 (15)	Cr2B—O7B—Cr1B	130.26 (10)
O3A—C3A—N5A	118.32 (16)	H1O1—O1W—H2O1	104 (2)
N6A—C3A—N5A	119.63 (15)		
			
Cr1A—O1A—C1A—N2A	−12.6 (3)	Cr1A—O5A—C5A—N9A	−33.6 (2)
Cr1A—O1A—C1A—N1A	167.40 (14)	Cr1A—O6A—C6A—N12A	−23.3 (3)
Cr1A—O2A—C2A—N4A	175.80 (14)	Cr1A—O6A—C6A—N11A	157.35 (14)
Cr1A—O2A—C2A—N3A	−2.8 (3)	O6B—Cr2B—O7B—Cr1B	−39.64 (15)
Cr1A—O3A—C3A—N6A	−26.1 (2)	O4B—Cr2B—O7B—Cr1B	83.00 (13)
Cr1A—O3A—C3A—N5A	154.40 (13)	O5B—Cr2B—O7B—Cr1B	−158.42 (12)
Cr1A—O4A—C4A—N8A	150.59 (14)	O3B—Cr1B—O7B—Cr2B	78.99 (14)
Cr1A—O4A—C4A—N7A	−29.9 (3)	O2B—Cr1B—O7B—Cr2B	−161.07 (13)
Cr1A—O5A—C5A—N10A	146.75 (14)	O1B—Cr1B—O7B—Cr2B	−41.89 (15)

### Hydrogen-bond geometry (Å, º)

**Table:**

*D*—H···*A*	*D*—H	H···*A*	*D*···*A*	*D*—H···*A*
N1*A*—H1*A*1···O2*B*^i^	0.87	2.42	3.051 (3)	129
N1*A*—H1*A*2···Br1*C*^ii^	0.87	2.60	3.4031 (18)	155
N2*A*—H2*A*1···O6*A*	0.87	2.20	2.890 (2)	136
N2*A*—H2*A*1···O1*W*	0.87	2.58	3.230 (3)	132
N2*A*—H2*A*2···Br1*C*^ii^	0.87	2.73	3.5121 (17)	150
N3*A*—H3*A*1···O3*A*	0.87	2.23	2.866 (2)	130
N3*A*—H3*A*2···O5*B*^iii^	0.87	2.21	2.972 (2)	147
N4*A*—H4*A*1···Br1*C*	0.87	2.55	3.4070 (18)	171
N4*A*—H4*A*2···O5*B*^iii^	0.87	2.11	2.900 (2)	151
N5*A*—H5*A*1···O6*B*^i^	0.87	2.52	3.174 (2)	133
N5*A*—H5*A*2···O3*B*^iv^	0.87	2.33	3.108 (2)	150
N6*A*—H6*A*1···O4*A*	0.87	2.17	2.914 (2)	143
N6*A*—H6*A*2···O1*B*^iv^	0.87	2.15	2.920 (2)	147
N7*A*—H7*A*1···O2*A*	0.87	2.12	2.865 (2)	143
N7*A*—H7*A*2···O4*B*^v^	0.87	2.24	3.092 (2)	168
N8*A*—H8*A*1···O7*B*	0.87	2.03	2.883 (2)	166
N8*A*—H8*A*2···O5*B*^v^	0.87	2.04	2.875 (2)	162
N9*A*—H9*A*1···O1*A*	0.87	2.22	2.954 (2)	141
N9*A*—H9*A*2···O6*B*^iv^	0.87	2.21	2.997 (2)	150
N10*A*—H10*A*···O1*B*	0.87	2.29	3.028 (2)	142
N10*A*—H10*B*···O4*B*^iv^	0.87	2.39	3.184 (2)	151
N11*A*—H11*A*···O1*W*	0.87	2.04	2.892 (3)	167
N11*A*—H11*B*···Br1*C*^vi^	0.87	2.57	3.4031 (18)	162
N12*A*—H12*A*···O5*A*	0.87	2.27	2.982 (2)	140
N12*A*—H12*A*···O2*B*	0.87	2.54	3.051 (2)	118
N12*A*—H12*B*···Br1*C*^vi^	0.87	2.85	3.6242 (18)	149
O1*W*—H1*O*1···Br1*C*	0.85 (1)	2.51 (1)	3.332 (2)	163 (3)
O1*W*—H2*O*1···O5*B*^vii^	0.84 (1)	2.57 (2)	3.315 (3)	149 (3)

Symmetry codes: (i) *x*, *y*−1, *z*; (ii) −*x*+1, −*y*, −*z*+1; (iii) −*x*+3/2, *y*−1, −*z*+3/2; (iv) −*x*+1, −*y*+1, −*z*+2; (v) −*x*+3/2, *y*, −*z*+3/2; (vi) −*x*+1, −*y*+1, −*z*+1; (vii) *x*−1/2, −*y*+1, *z*−1/2.
